# Effect of preoperative sleep disorders on delirium in proximal femoral surgery patients aged 60 or older

**DOI:** 10.1186/s12871-023-02331-6

**Published:** 2023-11-17

**Authors:** Fuyi Han, Xiaojie Liu, Hui Huang, Haichen Chu, Wei Feng

**Affiliations:** 1 Department of Anesthesiology, The Fourth Affiliated Hospital, Zhejiang University School of Medicine, Yiwu, 322000 Zhejiang China; 2 Department of Anesthesiology, The Affiliated Hospital of Qingdao University, Qingdao, 266003 China

**Keywords:** Sleep disorder, Hip fractures, Postoperative delirium

## Abstract

**Objective:**

To examine the effect of preoperative sleep disorders on delirium in patients older than 60 years of age who underwent surgery for proximal femoral fracture.

**Methods:**

This is a prospective observational study. We prospectively selected 143 patients with proximal femoral fracture who underwent surgery between April 2021 and April 2022. The primary outcome was postoperative delirium (PD). Multiple logistic regression analyses were performed and a receiver operating characteristic (ROC) curve was generated. The preoperative sleep quality of all eligible participants was assessed through the Pittsburgh Sleep Quality Index (PSQI). The Confusion Assessment Method (CAM) was used to assess PD from the first to the seventh day postoperatively. Patients were divided into two groups according to the PD diagnosis: (1) the no PD (NPD) group and (2) the PD (PD) group.

**Results:**

Of 143 eligible patients, 43 (30.1%) were diagnosed with PD. Multiple logistic regression analysis demonstrated that postoperative ICU admissions (OR = 2.801, *p* = 0.049) and preoperative sleep disorders (OR = 1.477 *p* < 0.001) were independently associated with PD. A receiver operating characteristic (ROC) curve demonstrated that the preoperative PSQI score was predictive of PD (AUC 0.808, 95% CI 0.724 ~ 0.892, *p* < 0.001).

**Conclusion:**

Preoperative sleeping disorders may be an independent risk factor leading to PD and an independent predictive factor for the development of delirium in proximal femoral surgery patients aged 60 or older.

## Introduction

As bone mass decreases with age, the risk of fracture increases. Although hip fractures are not the most common fracture in elderly individuals, they have become the most feared fracture because hip fractures can have serious complications throughout the perioperative period [1, 2].

As one of the most common complications in elderly patients with proximal femoral fracture, the incidence of postoperative delirium varies from 4 to 53.3% [3]. PD is an acute, fluctuating state of brain dysfunction caused by multiple mechanisms, characterized by inattention, confusion, and an altered state of consciousness, which usually occurs 3 days after surgery [4, 5]. PD is a risk factor for a variety of short-term and long-term complications. It can affect physical and cognitive recovery and significantly increase medical and nursing costs. The onset of delirium depends on a complex interplay between the patient’s baseline vulnerability and predisposing factors that occur during hospitalization. It is known that risk factors for delirium are 65 years of age or older, male sex, dementia, depression, American Society of Anesthesiologists physical status classification (ASA classification), operation type, visual impairment, body mass index (BMI), preoperative serum albumin and hemoglobin level, fractures to the surgery time interval, medication, intraoperative blood pressure, temperature, depth of anesthesia, blood transfusion, and postoperative analgesia [6–11].

Sleep disorders are common among the elderly in China, especially among elderly females, with a prevalence of 35.9%. A small retrospective cohort study showed that sleep disorders prior to surgery were significantly associated with the development of delirium in patients aged 60 years and older with proximal femoral fractures [12]. However, no studies have evaluated the effect of preoperative sleep disorders on PD in elderly patients with proximal femoral fractures undergoing surgery. Here, we hypothesized that preoperative sleep disorders may be a risk factor for delirium. Therefore, the aim of this study was to investigate the relationship between preoperative sleep disorders and delirium development in elderly patients with proximal femoral fractures undergoing surgery.

## Methods

This study is a prospective observational cohort study. The study was approved by the Ethics Committee of the Affiliated Hospital of Qingdao University (QYFYWZLL27312) and is consistent with the Declaration of Helsinki. All patients provided written informed consent. The inclusion criteria were as follow: (1) age ≥ 60 years and (2) proximal femoral surgery. The exclusion criteria included: (1) having a history of delirium or dementia; (2) having a history of brain trauma; and (3) being unable to communicate due to hearing and/or language issues.

All patients underwent surgery by general anesthesia with the same anesthesia program. A bispectral index (BIS) was used to monitor the depth of anesthesia, and BIS values were maintained between 40 and 60 during surgery. A normal body temperature was maintained, and anticholinergics were conserved perioperatively because of the possible occurrence of latent psychiatric disorders.

Preoperative sleep quality was assessed by the Pittsburgh Sleep Quality Index (PSQI). The PSQI has been indicated to be an effective way to assess sleep quality [13]. It is divided into 7 sections with a total of 18 items, with scores ranging from 0 to 21. A PSQI score of 5 is considered the cutoff point for sleep disorders in adults. The higher the PSQI score is, the more severe the sleep disorders.

The Confusion Assessment Method (CAM) recommended by the European Society of Anesthesiology was used to determine whether PD occurred [14]. Among them, the Confusion Assessment Method for Intensive Care Unit (CAM-ICU) is widely used to evaluate delirium in the intensive care unit (ICU) [15]. The CAM-ICU is divided into four sections: (1) acute changes in mental status and behavior over the past 24 h; (2) inattention; (3) disordered thinking; and (4) an altered state of consciousness. Delirium is diagnosed when items (1), (2) and (3) or (4) are met. In addition, delirium was assessed only once within 24 h after surgery and as close to 24 h after surgery as possible to minimize the impact of anesthesia on the patient’s consciousness. On postoperative Days 2–7, the patients were evaluated twice a day (8:00–10:00 and 18:00–20:00). All perioperative data were evaluated and recorded by the same anesthesiologist who did not participate in anesthesia and did not know the grouping.

All participants received intravenous sufentanil and dezocine to achieve a perfect analgesic score (numeric rating scale, NRS < 4). If necessary, dexmedetomidine may be injected to control delirium onset [16].

The variables included sex, age, BMI, ASA classification, preoperative PSQI score, preoperative albumin level, preoperative hemoglobin level, intraoperative blood loss, fractures to the surgery time interval, anesthesia duration, operation time, operation type, diabetes, hypertension, stroke, coronary heart disease, postoperative ICU admissions and postoperative delirium. We divided all participants into two groups based on the diagnosis of PD: (1) the no PD (NPD) group and (2) the PD (PD) group.

### Statistical analysis

The normality of the distribution of continuous variables was determined by the Kolmogorov‒Smirnov test. Continuous variables with a normal distribution are presented as the mean ± standard deviation (SD), and variables with an abnormal distribution are presented as the median, interquartile range (IQR) and range. Categorical variables are expressed as numbers and percentages. Student’s t test and the Mann‒Whitney U test were used for continuous variables, and the Pearson chi‒square test was used for categorical variables.

Simple logistic regression was used to analyze the relationship between demographic and clinical data and PD. An index of *p* < 0.1 was incorporated into the multivariate regression analysis. Multivariate logistic regression models were used to analyze the association between PD and covariates found to be associated with delirium in univariate analyses. Statistical significance was set as a *p* value of less than 0.05. SPSS 23.0 (IBM Corp, Armonk, NY) was used to analyze the data.

The ROC curve was drawn to further determine the accuracy, sensitivity and specificity of the model. A *p* value of less than 0.5 was considered to indicate that the model had certain predictive value.

## Results

**Fig. 1:**
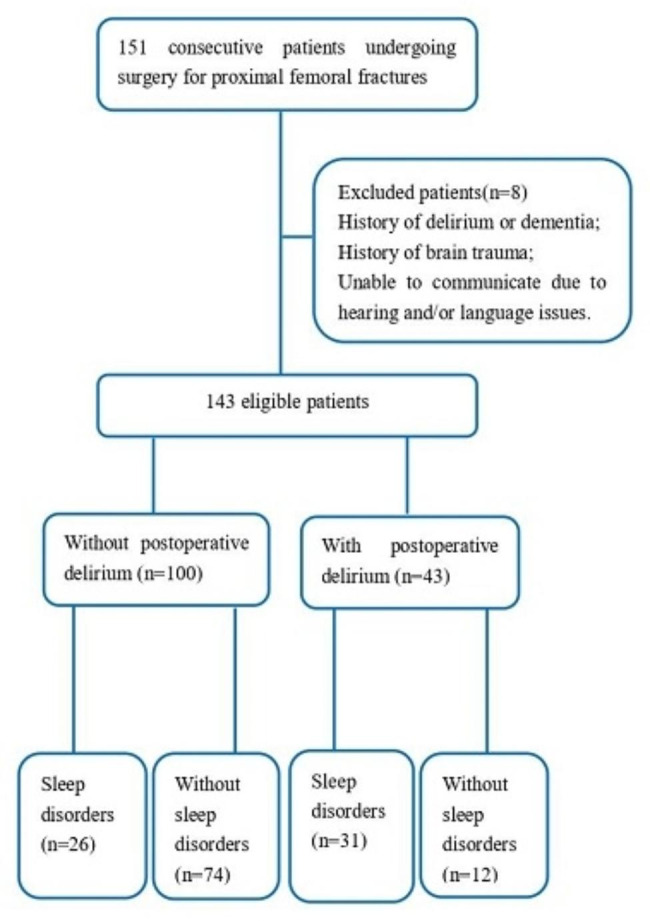
The flow chart of screening cases for this study

A total of 151 patients who underwent surgery for proximal femoral fractures from April 2021 to April 2022 were enrolled. Eight patients were excluded according to the exclusion criteria. Of these, 4 patients had a history of delirium or dementia, 2 patients had a history of brain trauma, and 2 patients were unable to communicate due to hearing and/or language issues. A total of 143 patients were divided into two groups according to PD. Among the 100 patients without PD, 26 had preoperative sleep disorders, and among the 43 patients with PD, 31 had preoperative sleep disorders. No patient died or dropped out during follow-up (Fig. 1).

Univariate analyses were performed for baseline values and general characteristics. Risk factors associated with PD included: ASA classification, age, albumin level, hemoglobin level, preoperative PSQI score, operation type, coronary heart disease and postoperative ICU admissions (*p* < 0.05) (Table 1).

**Table 1:** Univariate analysis according to diagnosis of postoperative delirium

	NPD(n=100)	PD(n=43)	*p*
	75.9 ± 8.7	81.4 ± 7.3	<0.001
BMI (kg/m^2^), medians (IQRs)	23.1(21.3,26.2)	23.7(21.7,25.4)	0.65
ASA classification, n (%)			0.006
I	12(12.0)	2(4.7)	
II	76(76.0)	26(60.5)	
III	12(12.0)	15(34.9)	
Female, n (%)	70 (70.0)	31(72.1)	0.801
Albumin level (g/l), (mean ± SD)	34.7 ± 3.8	32.6 ± 4.2	0.003
Hemoglobin level (g/l), (mean ± SD)	114.1 ± 21.1	105.8 ± 16.5	0.025
Fractures to the surgery time interval (day), medians (IQRs)	5.0(3.0,8.0)	6.0(4.0,9.0)	0.128
Preoperative PSQI score, (mean ± SD)	4.3 ± 2.7	9.4 ± 4.8	< 0.001
Operation type, n (%)			0.045
Total hip arthroplasty	25(25.0)	3(7.0)	
Bipolar hemiarthroplasty	26(26.0)	14(32.6)	
Internal fixation	49(49.0)	26(60.5)	
Operation time (min), (mean ± SD)	119.9 ± 52.2	123.3 ± 51.0	0.719
Anesthesia duration (min), (mean ± SD)	176.9 ± 54.4	175.6 ± 48.9	0.891
Intraoperative blood loss (ml), (mean ± SD)	182.2 ± 146.8	159.3 ± 92.7	0.348
Hypertension, n (%)	50 (50.0)	24 (55.8)	0.523
Coronary heart disease, n (%)	22 (22.0)	18 (41.9)	0.015
Diabetes, n (%)	32 (32.0)	14 (32.6)	0.948
Stroke,n (%)	9(9.0)	7(16.3)	0.329
Postoperative ICU admissions, n (%)	30(30)	30(69.8)	< 0.001

NPD, no postoperative delirium; PD, postoperative delirium; SD, standard deviation; PSQI, Pittsburgh sleep quality index; ICU, intensive care unit

Multiple logistic regression analysis demonstrated that postoperative ICU admissions (OR = 2.801, p = 0.049) and preoperative sleep disorders (OR = 1.477, p < 0.001) were independently associated with PD (Table 2).

**Table 2:** Logistic regression for postoperative delirium as an independent variable

	Univariate analyses		Multivariate analyses	
	Crude OR (95% CI)	*p*	Adjusted OR* (95% CI)	*p*
Preoperative PSQI score	1.416(1.252–1.602)	<0.001	1.477(1.264–1.727)	<0.001
Age	1.081 (1.033–1.131)	0.001	1.065(0.993–1.143)	0.080
Albumin level	0.868(0.788–0.955)	0.004	0.871(0.758–1.001)	0.052
Hemoglobin level	0.980(0.962–0.998)	0.351		
Operation type				
Total hip arthroplasty	Ref		Ref	
Bipolar hemiarthroplasty	4.487 (1.149–17.526)	0.031	2.564(0.426–15.419)	0.304
Internal fixation	4.422 (1.225–16.044)	0.024	2.625(0.520–13.251)	0.243
Coronary heart disease	2.553 (1.183–5.516)	0.135		
ASA classification				
I	Ref		Ref	
II	2.053 (0.431–9.785)	0.367	5.161(0.452–58.946)	0.187
III	7.500 (1.400–40.178)	0.019	1.103(0.304–4.005)	0.882
Postoperative ICU admissions	6.028(2.731–13.306)	<0.001	2.801(1.004–7.811)	0.049

*Adjusted by Albumin level, Postoperative ICU admissions.

**Fig. 2:**
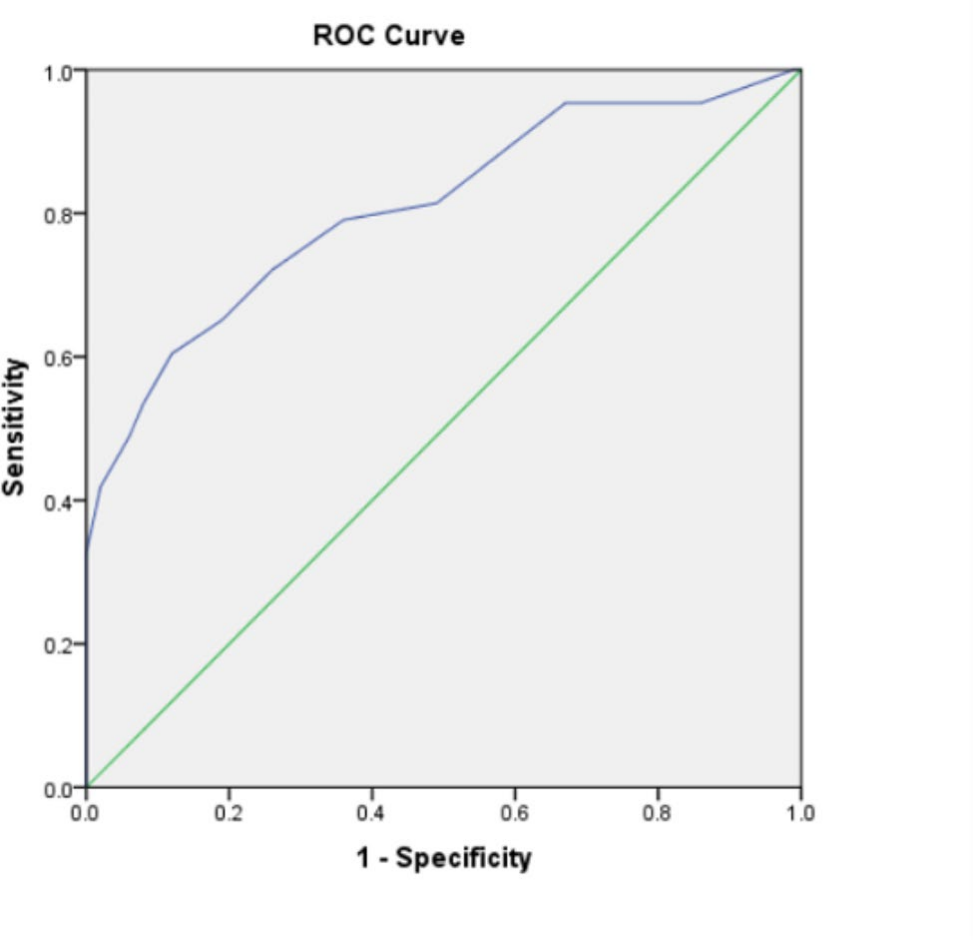
Receiver operating characteristic (ROC) curve of preoperative Pittsburgh sleep quality index (PSQI) score of predictability for postoperative delirium. (Fig. 2)

The ROC curve demonstrated that the preoperative PSQI score was predictive of PD (AUC 0. 808, 95% CI 0.724–0. 892, p < 0.001) (Fig. 2). The cutoff value was 7.5, with a sensitivity of 60% and a specificity of 88%(Fig. 2).

## Discussion

This study illustrated that preoperative sleep disorders were independently correlated with PD, in addition to the usual risk factors.

Delirium is frequently encountered in the ICU. Precipitating factors related to the treatment include: respiratory failure, shock, metabolic disturbances, prolonged mechanical ventilation, pain, immobility, sedatives and adverse environmental conditions impairing vision, hearing, and sleep [17]. Postoperative delirium was also associated with baseline conditions in ICU patients [18]. The management of ICU delirium has historically been challenging, as very few pharmacological options have demonstrated efficacy in treating delirium once it develops. However, it is difficult to ensure that the anesthesiologist involved in the assessment of a patient’s postoperative delirium is completely unaware of whether the patient is an ICU patient, because the conscious state of the patient admitted to the ICU determines the need for the patient to use the ICU-CAM to assess delirium. ICU admission as a risk factor may therefore be biased.

Evidence suggests that undernutrition negatively impacts health-related quality of life and postoperative outcomes [19, 20]. Elderly patients with proximal femoral fracture are often prone to metabolic disorders, low calorie consumption, impaired nutrition and concomitant cerebrovascular disease leading to impaired nutritional intake [21]. However, there was no significant difference in BMI or the Albumin level between the two groups. A larger sample size may be needed.

Risk factors for PD can be roughly divided into two categories: predisposing factors and precipitating factors. The predisposing factors include advanced age, dementia, cognitive impairment, multiple medical diseases, hearing and visual impairment, and history of alcohol consumption. The precipitating factors include pain, depression, anemia, infection, malnutrition, activity limitation, hypoxemia, dehydration and electrolyte disturbances, acid-base imbalance, urinary retention and constipation, medications (anticholinergic drugs, etc.) and sleep disorders.

The mechanisms by which sleep disorders cause cognitive and behavioral dysfunction are not well understood. Both anxiety, depression and sleep apnea (OSA) can lead to sleep disorders. Chronic insomnia caused by anxiety and depression is associated with impairment of alertness, orientation, and behavioral control [22, 23]. A previous study found that chronic intermittent hypoxemia and hypercapnia that result from OSA can also impair attention, memory, and cognition [24]. It has been reported that a large amount of cortisol production induced by hypothalamic‒pituitary‒adrenal axis hyperexcitability is transferred to the central nervous system, leading to apoptosis of neurons in the hippocampal area through activation of the brain-derived neurotrophic growth factor-tropomyosin-related kinase B signaling pathway, which may be a possible mechanism that explains how sleep disorders influence cognitive and behavior dysfunction [25]. Another study illustrated that hypoxemia and hypercapnia caused by long-term OSA can also damage vascular endothelial cells, improve the sensitivity of the vasoconstrictive response, produce an oxidative stress response leading to a hypercoagulable state, and increase the risk of vascular dementia [26]. Moreover, another study found that neuronal apoptosis induced by sleep disorders, related to autonomic nervous system imbalance, may also be the mechanism. Therefore, the relevance of studying the impact of preoperative sleep quality on PD for patients is clear.

In the current study, we found that ASA classification, age, preoperative albumin level, preoperative hemoglobin level, operation type, preoperative coronary heart disease, and postoperative ICU admission may be risk factors associated with PD through univariate analysis. However, none were determined because of their interaction with each other. We used multiple logistic regression models to adjust for covariates, and the models showed that preoperative sleep disorders and postoperative ICU admission were reliable independent risk factors.

The AUC generated from the ROC curve can predict the diagnostic value of a risk factor for the outcome. An AUC greater than 0.7 means that the risk factor has a certain accuracy in predicting the outcome. The ROC curve was used to observe the effect of the preoperative PSQI score on the incidence of PD, and the AUC obtained was 0.808, indicating that the preoperative PSQI score is a diagnostic factor for PD. The results showed that the PSQI had certain diagnostic value for PD.

Our study has some advantages in demonstrating that preoperative sleep disorders are associated with PD. First, prospective observational studies can avoid bias caused by retrospective studies. Second, the preoperative sleep quality of patients was assessed by the PSQI, which has a sensitivity of 89.6% and a specificity of 86.5% for the judgment of sleep quality. It can analyze sleep disorders caused by anxiety, depression and breathing problems, and describe sleep conditions comprehensively and specifically. Third, the program of general anesthesia was standardized to decrease the possibility of a narcotic administration bias.

This study has several limitations. First, since this study included elderly patients, subacute delirium may be misdiagnosed as a deconditioning postoperative event. Delirium could be underestimated when symptoms involve hypoactive conditions. We are unsure of how a higher incidence of PD could influence the outcome. Second, the PSQI and CAM are self-report questionnaires for sleep quality and delirium, so there must be some bias in the diagnosis of SPD and PD. Polysomnography, electroencephalography and blood tests may help. Third, this study has a small sample size and a short research time. Studies with larger sample sizes are needed.

## Conclusions

Preoperative sleeping disorders are an independent risk factor leading to PD and an independent predictive factor of delirium in proximal femoral fracture patients aged 60 or older.

## Data Availability

The datasets used and analyzed during the current study are available from the corresponding author upon reasonable request.
